# Immunocytochemical localization of saikosaponin-d in vegetative organs of *Bupleurum scorzonerifolium* Willd

**DOI:** 10.1186/1999-3110-54-32

**Published:** 2013-09-10

**Authors:** Xueyan Zhao, Li Zheng, Jingjing Si, Yan Miao, Yong Peng, Xia Cai

**Affiliations:** Key Laboratory of Resource Biology and Biotechnology in western China, (Northwest University), Ministry of Education, Xi’an, 710069 China

**Keywords:** *Bupleurum scorzonerifolium* Willd, Immunocytochemical localization, Saikosaponin-d, Vegetative organs

## Abstract

**Background:**

Saikosaponin-d (SSd) is an important active component of *Bupleurum scorzonerifolium* Willd., a traditional Chinese medicinal herb. Thus far, the biosynthetic pathway and biosynthetic site of saikosaponins in *Bupleurum* are largely unknown. The cellular localization of SSd will help in understanding saikosaponin biosynthesis and regulation.

**Results:**

In this study, we characterize for the first time the localization of SSd in *B. scorzonerifolium* tissues and cells using histochemistry and immunoelectron microscopy. The results show that the saikosaponin distribution in different plant organs changes as they mature. The number of SSd gold particles distinctly differed among the roots, stems, and leaves, with the particles mainly concentrated in the roots. The gold particles were mainly observed in vacuoles, with a few particles in the protoplasm; hence, SSd is mainly stored in vacuoles.

**Conclusions:**

We speculate that saikosaponins are mainly synthesized via the mevalonate pathway in the protoplasm in young organs, and then transported to the central vacuole by the endoplasmic reticulum (ER) or the fusion of vacuoles, to protect plants from self-poisoning with the accumulation of more saikosaponins.

**Electronic supplementary material:**

The online version of this article (doi:10.1186/1999-3110-54-32) contains supplementary material, which is available to authorized users.

## Background

*Bupleurum scorzonerifolium* Willd of family Umbelliferae is also called Shannon Chaihu, soft Miller Chaihu, south Chaihu. It has been a well-known traditional herbal medicine in China for more than a thousand years. The Chinese Pharmacopoeia lists *B. scorzonerifolium* root as the crude drug of Radix Bupleuri (Pharmacopoeia Commission of People’ s Republic of China 2010 edition). Saikosaponins, a group of triterpene saponins, are the major active constituents of *B. scorzonerifolium* and its related pharmacological effects (Bao et al. 2004; Xie et al. 2007; Jia and Zhang 1989). Up to 90 saponin compounds have been isolated from Radix Bupleuri (Xie et al. 2007), with saikosaponins a, c, and d constituting the highest contents. Pharmacologic experiments have shown that saikoponin a and saikoponin d have obvious pharmacologic activity, and their contents have become the standards for the quality control of medicinal samples (Bao et al. 2004). Saikosaponin d (SSd) is a triterpenoid saponin monomer that has anti-inflammatory and endocrine-regulating properties (Xie et al. 2007). SSd also inhibits Na-ATPase activity and it has a potential anti-tumor effect because of its glucocorticoid-like steroid ring structure (Jia and Zhang 1989).

Most studies on *Bupleurum* have mainly focused on component analysis, pharmacology, chemical structure, and clinical applications (Tan et al. 2008; Kanazawa et al. 1990; Guo et al. 1996; Ebata et al. 1996; Park et al. 2000). Consequently, the saikosaponin biosynthetic pathway and site in *Bupleurum* are unknown, although some studies have reported the cloning of critical gene fragments and gene regulation in the triterpene biosynthetic pathway of *Bupleurum* (Kim et al. 2006; Kim et al. 2011; Dong et al. 2011; Sui et al. 2010).

We previously determined the accumulative and distributive patterns of saikosaponins in *B. scorzonerifolium* root using lead acetate precipitation and transmission electron microscopy (Cai et al. 2009). However, we were unable to determine the cellular localization of SSd, which is crucial for further studies on the biosynthesis and regulation of saikosaponins.

Faulk and Taylor (1971) were the first to use colloidal gold–labeled antibodies and they achieved satisfactory results. Since then, gold-labeling technology has been used widely in studies on the distribution of polypeptides, acids, and auxins in plants. In this study, we first used the histochemical method to localize saikosaponins in vegetative organs and then adopted colloidal gold immunoelectron microscopy to localize SSd at the ultrastructural level to provide a basis for further studies on saikosaponin biosynthesis and transport in plant cells.

## Methods

### Materials

*Bupleurum scorzonerifolium* Willd. samples were collected from healthy plants grown in the field at the Botanical Garden of Northwest University in Shaanxi Province (Shaanxi, PR. China).

### Histochemical method

Fresh roots, stems, and leaves of *B. scorzonerifolium* were cut into 30 μm to 40 μm sections on a Leica CM 1850 cryostat microtome and stained with 5% vanillin–glacial acetic acid–perchloric acid solution. The sections were observed under a Leica DMLB microscope. The control materials were fixed in formalin-acetic acid-alcohol (FAA) for one month to remove the saikosaponins. After washing, the sections were stained and were examined using the same method.

### SSd-BSA complete antigen preparation

SSd was conjugated with bovine serum albumin (BSA) to confer rabbits with immunity against SSd. Then, the carbodiimide (EDC) method was used.

### Rabbit polyclonal antiserum against SSd preparation

**Figure 1:**
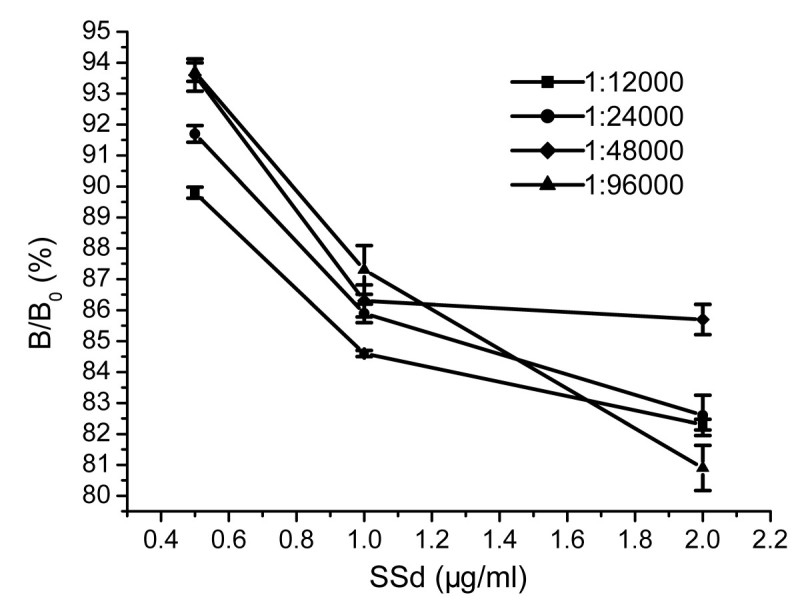
**Competitive ELISA curve of rabbit sera at different concentrations.** Coating antigen: SSd–BSA; blocked with 1% BSA; assay solution of 0.05 M carbonate buffer solution (pH 9.6); Color reagent: TMB.

The SSd–BSA was diluted to 600 μg/ml with PBS (0.1 M, pH 7.4). The solution was homogenized and heated in a 60°C water bath to improve solubility. Then, the solution was mixed with an equal volume of Freund’s complete adjuvant (Sigma), and thoroughly emulsified. The SSd–BSA adjuvant mixture (300 μg/each) was injected subcutaneously into the backs and the soles of three New Zealand white rabbits. Booster injections were given every two weeks. Blood samples were collected from the rabbits at 3 days after the fourth injection. The serum specimens were evaluated for the presence of anti-SSd antibodies using a competitive enzyme-linked immunosorbent assay (ELISA). The relative absorbance was calculated using the following formula:

$$\mathrm{Relative}\;\mathrm{absorbance}=B/B_{0},$$

where B_0_ is the absorbance of the serum without SSd and B is the absorbance of the serum with SSd. The corresponding standard curves (Figure 1) were constructed by plotting the average relative absorbance against the SSd concentration, with three replicates per concentration, using the Origin Program 6.0 software package. Figure 1 clearly shows the presence of SSd antibodies in the rabbit sera.

### Purification of SSd rabbit polyclonal antibodies

The serum was mixed 1:1 (v/v) with PBS (0.1 M, pH 7.4). The serum mixture was brought to 20% saturation with ammonium sulfate solution under continuous stirring at 4°C for 30 min. The mixture was centrifuged at 3000 × *g* for 20 min, and the pellet was discarded. Additional ammonium sulfate solution was added to the resulting supernatant to 50% saturation, and the suspension was stirred for 30 min at 4°C. The resulting precipitate was collected by centrifugation and dissolved in PBS (0.1 M, pH 7.4). Ammonium sulfate solution was added to the resulting supernatant to until 33% saturation. The suspension was stirred for 30 min at 4°C and centrifuged at 3000 × *g* for 20 min. This process was repeated 2–3 times. The resulting precipitate was resuspended in normal saline, and the mixture was dialyzed three times against PBS (0.1 M, pH 7.4) for 4 h each at 4°C. The dialyzed polyclonal antibodies were subjected to affinity chromatography on a *Staphylococcus* protein A Sepharose (SPA) column and a BSA affinity column.

### Preparation of colloidal gold-labeled SSd rabbit polyclonal antibodies

The purified SSd rabbit polyclonal antibodies were centrifuged at 100 000 × *g* for 60 min at 4°C, and the protein concentration of supernate was adjusted to 1 mg/ml. The pH of the colloidal gold solution was adjusted to 7.6. The protein solution was mixed with the colloidal gold solution and the mixture was brought to 0.05% saturation with 3% polyethylene glycol (PEG 20 000) under electromagnetic stirring. The mixture was centrifuged at 12 000 × *g* and the resulting pellet was resuspended in stabilizer solution. The gold-conjugated probe was stored at −20°C.

### Ultra-thin sectioning

Young and mature plant materials were divided into small pieces (0.5 mm × 0.5 mm × 0.5 mm) and fixed 2 h to 4 h in medium containing 2% paraformaldehyde and 0.5% glutaraldehyde at 4°C. After washing three times in PBS for 30 min, the materials were postfixed for 2 h in 1% osmium tetroxide in 0.1 M PBS (pH 7.4) at 4°C, and rinsed four times with distilled water (pH 7.0). The segments were dehydrated in a graded ethanol series and then embedded in Epon812.

Semithin sections (1 μm to 2 μm) were cut on a Reichert–Jung ultramicrotome and stained with methylene blue. The sections were examined under a Leica DMLB light microscope. Ultrathin sections (60 nm to 90 nm) were cut on an LKB-8000 II ultramicrotome, and collected on 200 mesh nickel grids with 0.3% Formvar.

### Immunostaining procedure

All subsequent steps were conducted at room temperature. The sections were first blocked for 20 min in TBS (pH 7.6) containing 1% rabbit serum, 0.05 M Tris–HCl and 0.1 M NaCl) to remove non-specific binding sites. The sections were incubated with the gold-conjugated probe for 20 min to 30 min, washed thoroughly with TBS, and rinsed with distilled water. Finally, they were stained with uranyl acetate and lead citrate.

The specificity of the gold-conjugated probe was assessed using the following control tests: (a) removing the SSd in the plant materials before preparing them for electron microscopy; (b) incubating the sections with the colloidal gold–labeled SSd antibodies containing excess SSd; and (c) incubating the sections without the colloidal gold–labeled SSd antibodies.

### Quantitative analysis of the density of gold particles

The density of gold particles in the selected area was measured as follows: photographic negatives at 20 000 × magnification were scanned and saved as figures. The areas on the figure that correspond to the protoplasm and vacuole were measured using Image-Pro Plus 6.0 software (Media Cybernetics, USA), and the number of gold particles in each cell region was counted. The labeling density is presented as the number of gold particles per μm^2^. In addition, a one-way ANOVA and a Duncan’s multiple range test were performed to investigate the differences in gold particle density between the protoplasm and the vacuole in cells from the same tissue at 0.05 probability levels using STATISTICA 6.0 software (Statsoft Inc., Tulsa, Oklahoma, USA).

## Results

### Histochemical localization of saikosaponin in vegetative organs

Saikosaponins react with certain chemical reagents to produce macromolecular complexes with characteristic colors; thus, increasing saikosaponin content shows characteristic colors from light red to purple red in 5% vanillin–glacial acetic acid–perchloric acid solution, which could be used to determine their distribution in the vegetative organs of *B. scorzonerifolium*.

**Figure 2:**
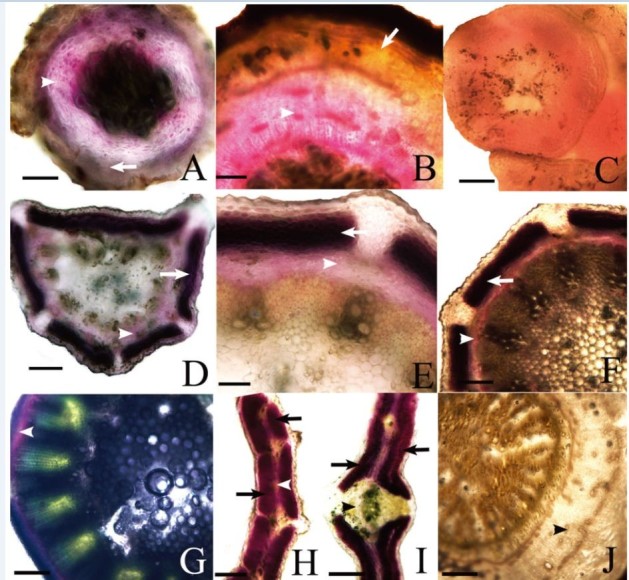
**The distribution of saikosaponins in vegetative organs of** ***Bupleurum scorzonerifolium*** **Willd. (A)** The pericycle (arrow) and primary phloem (arrowhead) of a 1-year-old root was stained red. **(B)** The phloem (arrowhead) and vascular cambium of the perennial root was stained purple red, whereas the pericycle (arrow) was stained light red. **(C)** The tissues of the shoot apex were stained light red. **(D–F)** showing the young stem, the cortex (arrow) showed dark purple red and the phloem (arrowhead) showed purple red. **(G)** In the mature stem, the phloem (arrowhead) was stained dark purple red. **(H–I)** The isobilateral leaf. **(H)** Structure of the blade: the palisade tissue (arrows) was stained purple red and the sponge tissue (arrowhead) showed light red. **(I)** The vein (arrowhead) in the blade remain unstained. **(J)** The control section of the root with the unstained phloem (arrowhead). Scale bars: A, B, F = 167 μm; C, I = 154 μm; D, E = 143 μm; G, H, J = 333 μm.

The pericycle and primary phloem of 1-year-old roots were stained red (Figure 2A), whereas in the perennial root, phloem, and vascular cambium were stained purple red (Figure 2B). All tissues in the shoot apex were stained light red (Figure 2C). The cortex of the primary and mature stems were stained dark purple red and the phloem was stained purple red (Figures 2D–G). The palisade tissues in mature leaves were stained dark purple red, the sponge tissues were stained light red, but the veins remained unstained (Figures 2H and I).

In the control experiment, the sections treated with FAA (formalin–acetic acid–70% alcohol) remained unstained (Figure 2J).

### Localization of SSd gold particles in the roots

**Figure 3:**
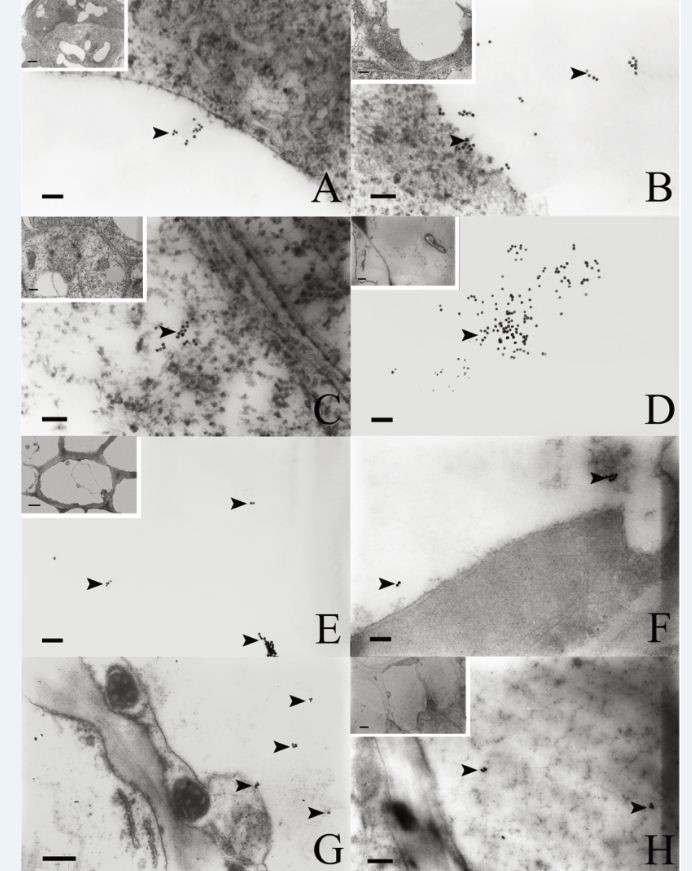
**Gold-labeling in the root.** Arrows indicated the gold particles: **(A)** Magnified view of a vacuole in a protoderm cell showing the gold-labeling; **(B)** Magnified view of a vacuole in a procambium cell showing the gold-labeling; **(C)** Magnified view of the protoplasm of a procambium cell showing the gold-labeling; **(D)** Magnified view of a vacuole in a cortical cell showing a large number of gold particles; **(E)** Vacuole in a primary phloem cell; **(F)** Many gold particles detected in the vacuole of a primary pericycle cell; **(G)** Large number of gold particles were labeled in the vacuole of a secondary phloem parenchyma cell. **(H)** Vacuole in a cambium cell. Scale bars: A–F = 50 nm; G = 200 nm; H = 100 nm.

The primary meristem cells in the *B. scorzonerifolium* root tip were compactly arranged, with thin walls, dense cytoplasm, and large nuclei. The protoderm cells contained a small number of gold particles, but only in the vacuoles (Figure 3A; Table 1). The ground meristem cells contained more gold particles than the protoderm cells, concentrated in very small vacuoles, with some particles in the protoplasm (Table 1). The procambium cells were faintly labeled in the vacuoles (Figure 3B) and protoplasm (Figure 3C, Table 1).

**Table 1:** **Differences in immunogold distribution among protoderm, procambium and ground meristem cells of root apex and shoot apex**

		Protoderm (n = 3)	Procambium (n = 3)	Ground meristem (n = 3)
Root apex	Protoplasm	-	1.26 ± 0.46b	3.04 ± 0.22b
	Vacuole	8.24 ± 1.65	3.22 ± 0.45a	10.21 ± 0.83a
Stem apex	Protoplasm	-	-	2.05 ± 0.36b
	Vacuole	-	-	3.17 ± 0.58a

1. Values are expressed as the mean number of gold particles per μm^2^*±* SE; 2. n = number of observations. 3. Different letters indicate significant differences in the density of gold particles between the protoplasm and the vacuole at the 0.05 significance level based on the Duncan’s Multiple Range Test.

The primary structure of root consists of the epidermis, cortex, and stele. The vacuoles of the epidermal cells contained a few gold particles; whereas the vacuoles of cortex cells contained a large number of scattered gold particles (Figure 3D). In the stele, the gold particles in the phloem cells exceeded those in the xylem cells, and they were mainly detected in vacuoles (Figure 3E and Table 2).

**Table 2:** **Differences in immunogold distribution among the roots, stems, and leaves**

		Young roots (n = 4)	Mature roots (n = 6)	Mature stems (n = 3)	Mature leaves (n = 4)
Cortex	Protoplasm	6.86 ± 1.00b	-	3.01 ± 0.60b	-
	Vacuole	18.31 ± 1.44a	-	5.12 ± 0.77a	-
Pericycle	Protoplasm	5.02 ± 1.39b	5.85 ± 0.97b	-	-
	Vacuole	21.06 ± 1.34a	17.91 ± 2.00a	-	-
Phloem	Protoplasm	4.35 ± 0.93b	12.50 ± 1.34b	1.39 ± 0.79b	-
	Vacuole	23.0 ± 2.17a	34.15 ± 2.03a	4.38 ± 0.34a	-
Cambium	Protoplasm	-	2.46 ± 1.22b	0.90 ± 0.47a	-
	Vacuole	-	19.47 ± 1.50a	1.22 ± 0.69a	-
Xylem	Protoplasm	-	-	1.60 ± 0.60a	-
	Vacuole	8.12 ± 1.22	13.75 ± 1.63	1.83 ± 0.56a	-
Pith	Protoplasm	-	-	-	-
	Vacuole	-	-	1.37 ± 0.62	-
Palisade tissue	Protoplasm	-	-	-	1.74 ± 0.90b
	Vacuole	-	-	-	6.10 ± 1.28a
Spongy tissue	Protoplasm	-	-	-	1.39 ± 0.61b
	Vacuole	-	-	-	4.41 ± 0.73a

1. Values are expressed as the mean number of gold particles per μm^2^ *±* SE; 2. n = number of observations

3. Different letters indicate significant differences in the density of gold particles between the protoplasm and the vacuole at the 0.05 significance level based on the Duncan’s Multiple Range Test.

The secondary structures of *B. scorzonerifolium* roots consist of the periderm, pericycle ring and secondary vascular tissue. The pericycle parenchymal cells had intercellular spaces and secretory canals composed of 3–4 epithelial cells. The pericycle cells and epithelial cells were rich in starch grains and osmiophilic droplets. The vacuole of pericycle parenchyma cells were labeled with numerous gold particles (Figure 3F and Table 2).

The secondary vascular tissue consists of secondary phloem, vascular cambium, and secondary xylem. Many gold particles were seen in vacuoles of the parenchymal cells in the secondary phloem (Figure 3G and Table 2), and a very few gold particles were detected in the intercellular space. The vascular cambium was formed by 3–5 closely arranged cell layers (Figure 3H), and numerous gold particles were observed in the vacuoles (Figure 3H and Table 2). The secondary xylem consisted of many parenchymal cells with scattered large vessels. A few gold particles were detected in the vacuoles of the parenchyma cells (Table 2).

### Localization of SSd gold particles in the stems

**Figure 4:**
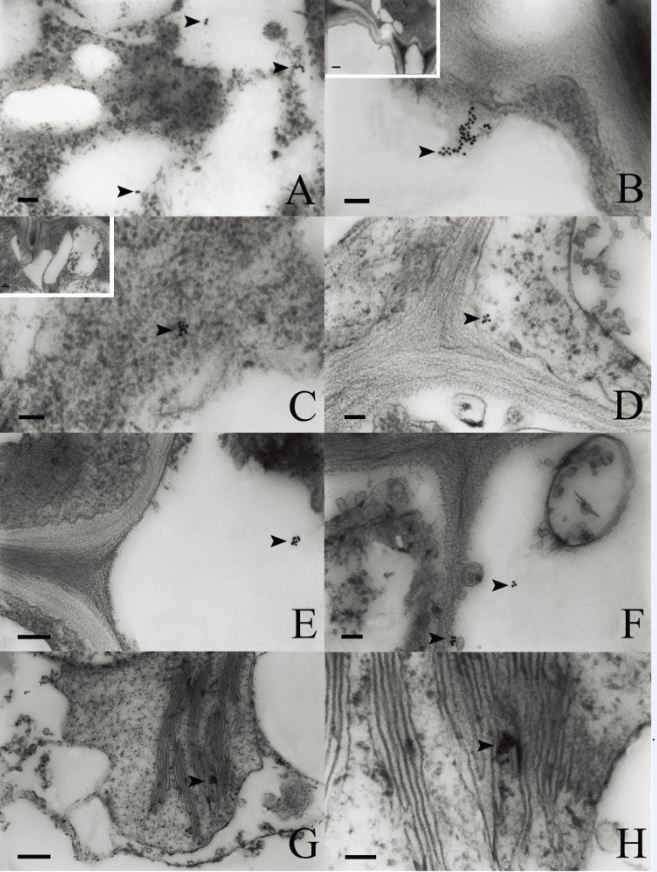
**Gold-labeling in the stems.** Arrows indicate the gold particles. **(A)** Gold particles were detected in the vacuoles of ground meristem cells in the shoot apex. **(B)** Magnified view of a vacuole in a cortex cell showing the gold-labeling. **(C)** Magnified view of the protoplasm of a cortex cell showing some gold particles. **(D)** Gold particles in the protoplasm of a phloem companion cell. **(E)** Gold particles in the vacuole of a secretory epithelial cell in the cortex. **(F)** Immunogold localization in the vacuole of a cambium cell. **(G)** Immunogold localization in the ER of a xylem parenchymal cell. *Bar* = 200 nm. **(H)** Magnified view of G. Scale bars: A–D, F, H = 50 nm; E = 100 nm; G = 200 nm.

The growing point of the stem was conical. The promeristem was composed of tunica cells and corpus cells compactly arranged with large nuclei and many small vacuoles. The primary meristem of the stem is formed through the division of derivative cells below the promeristem, which are composed of protoderm, procambium, and ground meristem cells. A few gold particles were observed in the vacuoles of the ground meristem cells (Figure 4A and Table 1). Then, the protoderm differentiates into the epidermis, the procambium into the vascular tissues, and the ground meristem into the cortex and pith, which form the primary structure. Under TEM, we found that the primary structure of stem had low saikosaponin levels. In the secondary structure, some gold particles were seen in the vacuoles (Figure 4B) and the protoplasm (Figure 4C) of the cortex cells (Table 2). A few gold particles were also detected in the protoplasm of companion cells (Figure 4D), in the vacuoles of epithelial cells of the secretory canal (Figure 4E), in the vacuoles of cambium cells (Figure 4F), and in the protoplasm of xylem parenchymal cells (Figures 4G and H and Table 2).

### Localization of SSd gold particles in leaves

**Figure 5:**
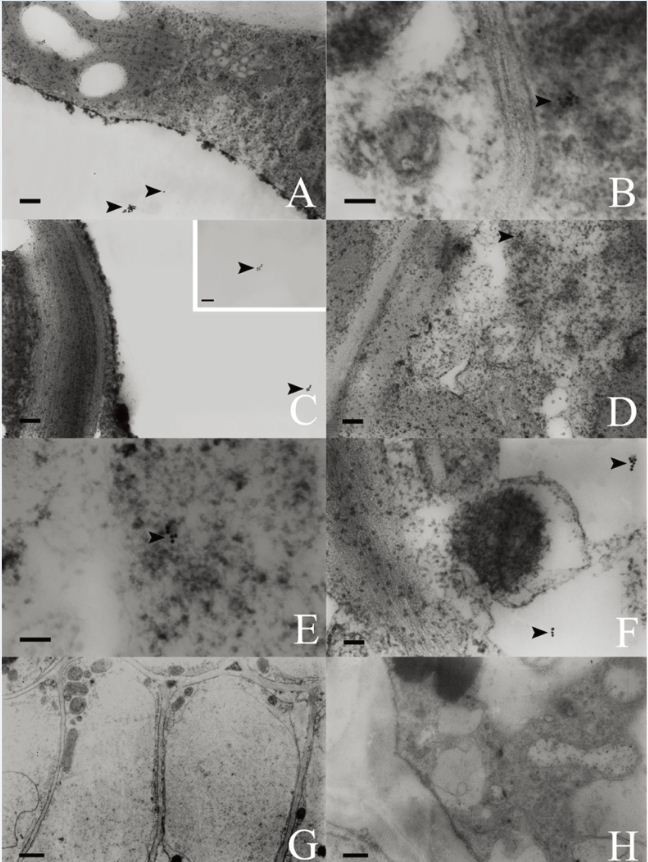
**Gold-labeling in the leaves.** Arrows indicate gold particles. **(A)** Gold-labeling in a vacuole of an epidermal cell. **(B)** Gold-labeling in the protoplasm of a meristem cell. **(C)** Gold particles in the vacuole of a meristem cell. **(D)** Gold-labeling in the protoplasm of a palisade parenchymal cell. **(E)** Magnified view of D. **(F)** Gold particles in the vacuole of a spongy parenchymal cell. **(G)** Control section wherein SSd was removed. **(H)** Control section incubated without colloidal gold–labeled SSd antibodies. Scale bars: A, C, D =100 nm; B, E, F = 50 nm; G = 500 nm; H = 200 nm.

In young leaves, the vacuoles in epidermal cells (Figure 5A), the protoplasm (Figure 5B) and the vacuoles in ground meristem cells were labelled with some gold particles (Figure 5C). The mature leaves of *B. scorzonerifolium* are typically isobilateral and they consist of the blade and the stipe. The blade consists of the epidermis, the mesophyll, and veins. The epidermal cells and vascular bundle cells of mature leaves were free of gold particles, and only a few gold particles were detected in the vacuoles and the protoplasm of palisade cells (Figure 5D and E) and spongy parenchymal cells (Figure 5F and Table 2).

Gold particles were rarely observed in the control sections wherein the SSd was removed (Figure 5G) and in the sections incubated without the colloidal gold–labeled SSd antibodies (Figure 5H).

## Discussion

The SSd in the roots, stems, and leaves of *B. scorzonerifolium* were examined through histochemistry and ultrastructural immunogold localization. The results show that saikosaponins distribution in the roots, stems, and leaves varied during different developmental stages. Tables 1 and 2 show the density of gold particles in selected areas of these organs. The level of saikosaponin labeling was higher in the vacuoles except the cambium and xylem cells in the stem. In addition, the density of gold particles differed significantly between the vacuoles and the protoplasm at the 0.05 significance level based on the Duncan’s Multiple Range Test. Consequently, the saikosaponins in the roots were mainly distributed in vacuoles of phloem cells. Shon et al. (1997) reported that the saikosaponins in *B. falcatum* are predominantly produced in phloem tissues regardless of cultivation year. Tan et al. (2008) reported that the saikosaponins in *B. chinense* are mainly distributed in the pericycle and primary phloem in young roots, but distributed in the vascular cambium and secondary phloem in mature roots. In mature stems, saikosaponins were mainly detected in the vacuoles of cortex cells. In mature leaves, saikosaponins were mainly found in vacuoles of palisade parenchyma cells. The results also indicate that saikosaponins mainly accumulate in the roots. Pan et al. (1984) found that saikosaponins are mainly distributed in phloem and ray cells, as well as in the xylem in the roots of eight medicinal *Bupleurum* species.

Colloidal gold, with its high electron density, is able to bind with a number of molecules, enabling it to become a non-radioactive tracer often used in fluorescent, radioisotope, and enzyme immunolabeling. In this study, we used immunolabeling to localize SSd for the first time. We found that the number of gold particles and the SSd content distinctly varied among the roots, stems, and leaves, with the roots containing several times more gold particles than the stems and leaves (Table 2). Therefore, the root has a higher SSd content than the stems and leaves, with the stems containing the lowest. This result is consistent with our previous result using biochemistry and phytochemistry (Tan et al. 2007). Tan et al. (2007) found that saikosaponins accumulate first in the roots, followed by the leaves, and finally the stems. Gan and Chen (1982) found that the saikosaponin content in the roots is 5.8-fold higher than those in the stems and in the leaves, and that more saikosaponin groups are present in the roots than in the stems and leaves. Wu et al. (2007) studied the transcription of the squalene synthase (SS) gene in the roots, stems, and rootstocks of *Panax notoginseng* and synthesis of triterpenoids. They found the total triterpenoids in the rootstocks are higher than in the leaves, followed by the roots, and the stems, probably because of differences in the expression of other key enzymes downstream of SS in the triterpenoid synthesis pathway or/and the directional transportation and accumulation of triterpenoids in different plant tissues and organs.

Saikosaponins are isoprenoids. They are derived from the universal five-carbon units isopentenyl diphosphate (IPP) and dimethylallyl diphosphate (DMAPP) through a series of catalytic reactions (Ramos-Valdivia et al. 1997; Sui et al. 2010). Terpenoids are biosynthesized via two pathways: the mevalonate (MVA) pathway and the methylerythritol phosphate (MEP) pathway. The typical MVA pathway occurs in the cytoplasm and ER (Chappell 1995), whereas the MEP pathway occurs in the plastids (Lange et al. 1998). Both pathways have isopentenyl diphosphate (IPP) as a major intermediate product. Isopentenyl diphosphate isomerase (IPI) is a core enzyme in isoprenoid biosynthesis (Ramos-Valdivia et al. 1997). Ramos-Valdivia et al. (1997) considered that IPIs are localized in various subcellular compartments and the localization patterns varied among different plant species. Nakamura et al. (2001) showed that IPI1 and IPI2 in *Nicotiana tabacum* are localized in the cytosol and plastids, respectively. Okada et al. (2008) reported that IPI proteins are mainly localized in the cytosol and mitochondria of *Arabidopsis*, and they played important roles in isoprenoid production via the MVA pathway. In most plants, IPI occurs in two different subcellular compartments, and it is essential for maintaining appropriate levels of IPP and DMAPP in different subcellular compartments (Chappell 1995; Nakamura et al. 2001; Okada et al. 2008). The present data reveals that SSd is distributed mainly in the vacuoles of the most parenchyma cells (Tables 1 and 2). The vacuole is the main repository of nutrients to protect cells and balance the nutrient supply (Mataunoto and Chung 1988; Femando et al. 1992). Moreover, it is a storage space for secondary materials that can enhance cell survival and competitiveness. Some plant cells store specific secondary compound in vacuoles to prevent self-poisoning (Renaudin and Guem 1988). Gold particles were also detected in protoplasm of some cells in the roots, stems, and leaves (Figures 4C and 5B), especially in the procambium cells in the roots (Figure 3C). They were also detected in the ER of xylem parenchyma cells (Figures 4G and H). Nevertheless, the immunoreactivity of the mature structures of three vegetative organs increased, especially in the roots (Tables 1 and 2). The saikosaponin biosynthesis likely occurs in the meristem, which has plenty of precursors but not saikosaponins; consequently, the meristem was poorly labeled with gold particles (Table 1). Hence, saikosaponins were mainly synthesized via the MVA pathway in the protoplasm in young organs, and then transported into vacuole by the ER or the fusion of vacuoles, to protect plants from self-poisoning through the accumulation of more saikosaponins.

## Conclusions

The localization of SSd in *B. scorzonerifolium* tissues and cells was studied using histochemistry and immunoelectron microscopy for the first time. The results showed that SSd was mainly stored in vacuoles. SSd were also detected in the ER. The immunoreactivity of the mature structures of three vegetative organs increased, especially in the roots. As a result, it was speculated that saikosaponins are mainly synthesized via the mevalonate pathway in the protoplasm in young organs, and then transported to the central vacuole by the ER or the fusion of vacuoles.

## Authors’ original submitted files for images

Below are the links to the authors’ original submitted files for images. Authors’ original file for figure 1 Authors’ original file for figure 2 Authors’ original file for figure 3 Authors’ original file for figure 4 Authors’ original file for figure 5
