# Supplementary figures and images for: Immunocytochemical localization of saikosaponin-d in vegetative organs of Bupleurum scorzonerifolium Willd
Source: Bot Stud. 2013 Sep 10;54:32. doi: 10.1186/1999-3110-54-32 (PMC5430319; doi:10.1186/1999-3110-54-32)

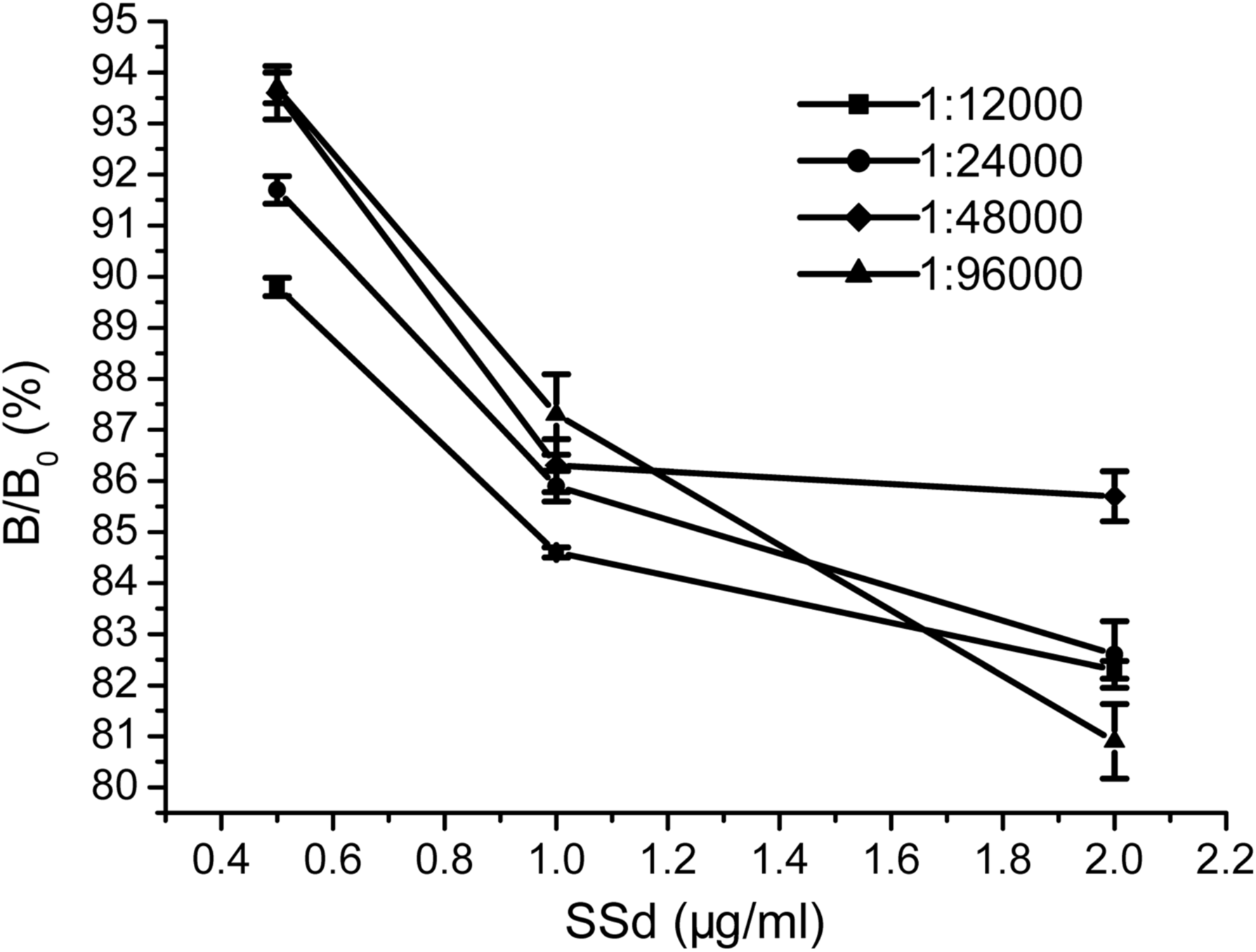

Supplement: Supplementary file 1 — Authors’ original file for figure 1 [file 40529_2013_25_MOESM1_ESM.tif]

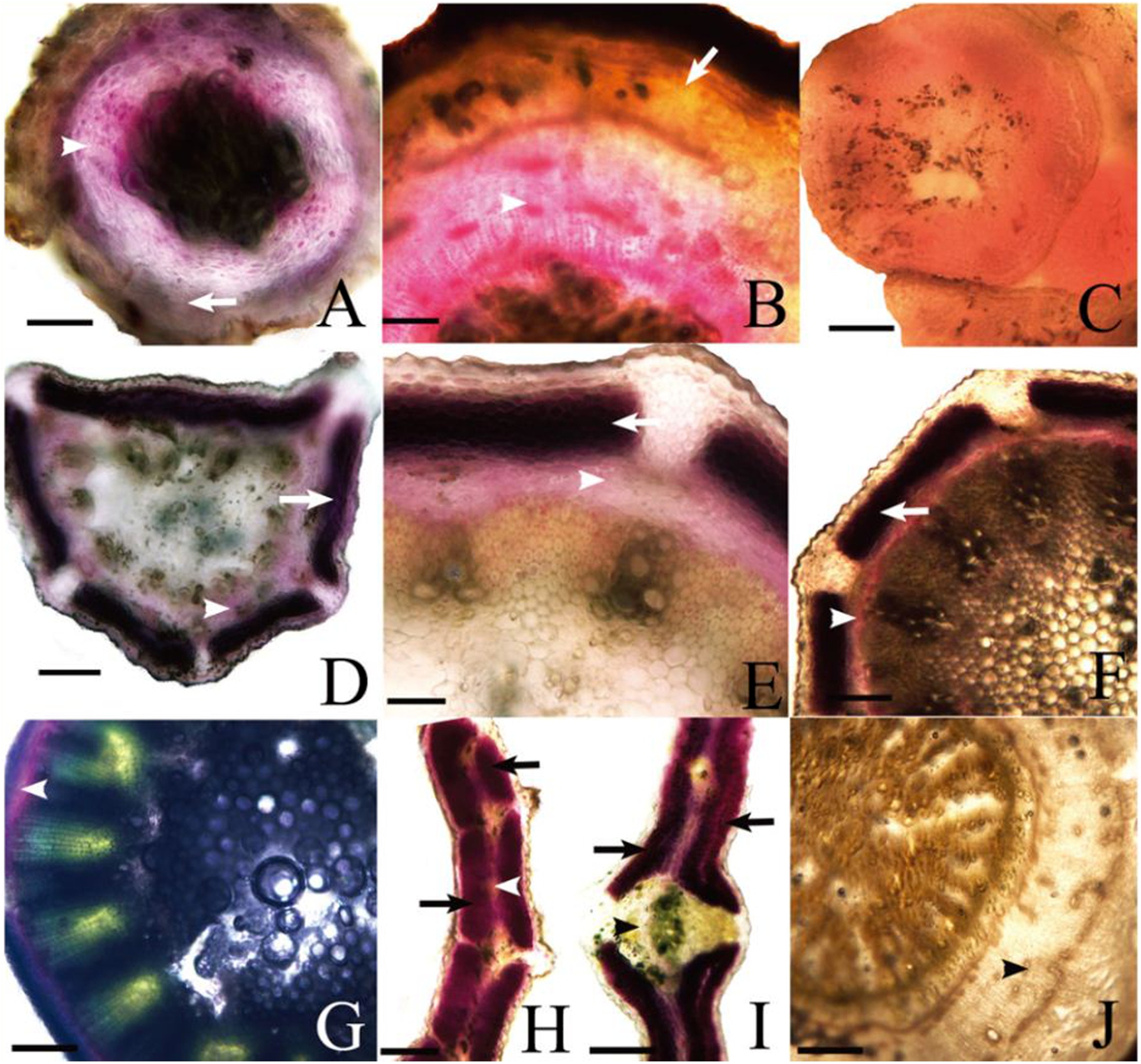

Supplement: Supplementary file 2 — Authors’ original file for figure 2 [file 40529_2013_25_MOESM2_ESM.tif]

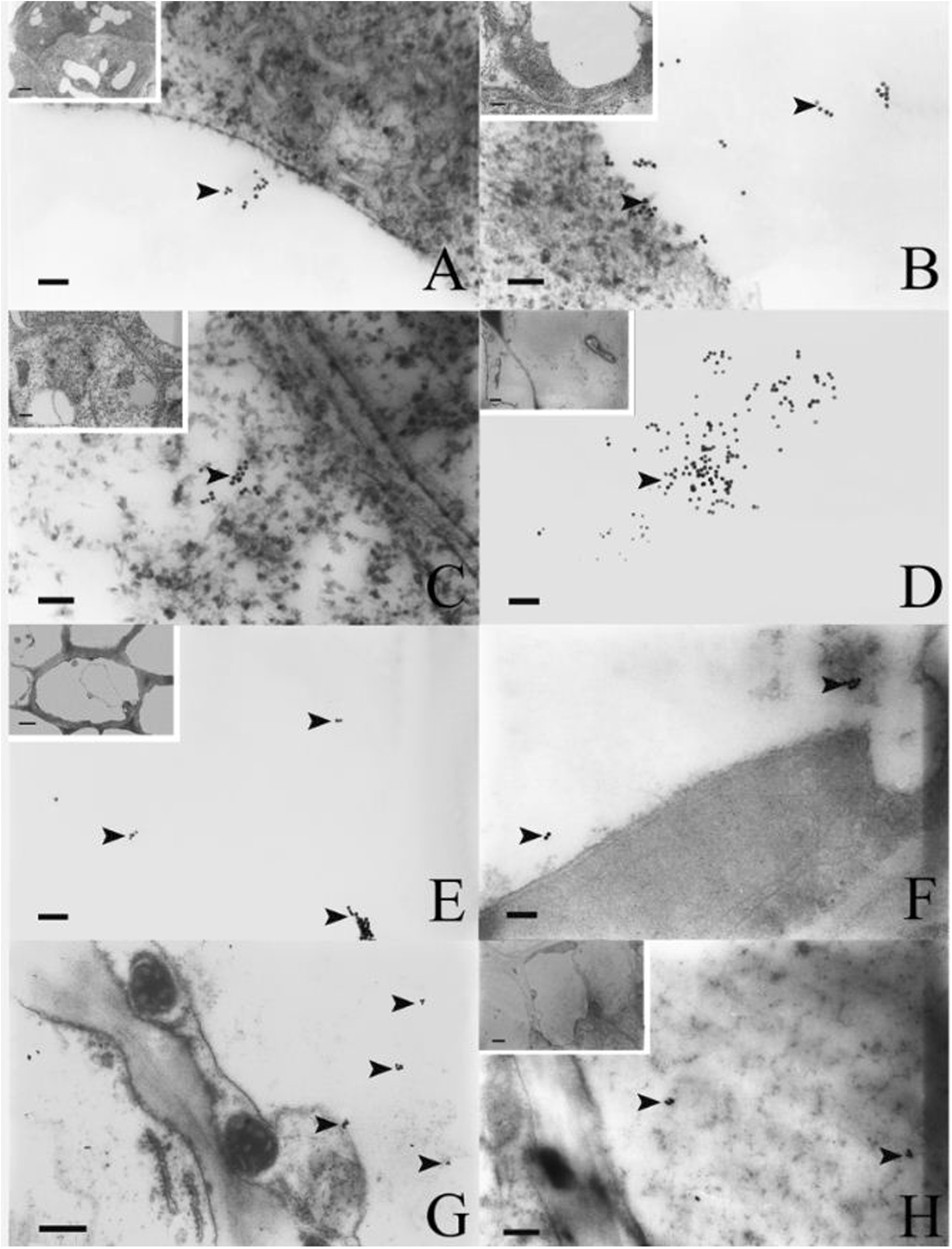

Supplement: Supplementary file 3 — Authors’ original file for figure 3 [file 40529_2013_25_MOESM3_ESM.tif]

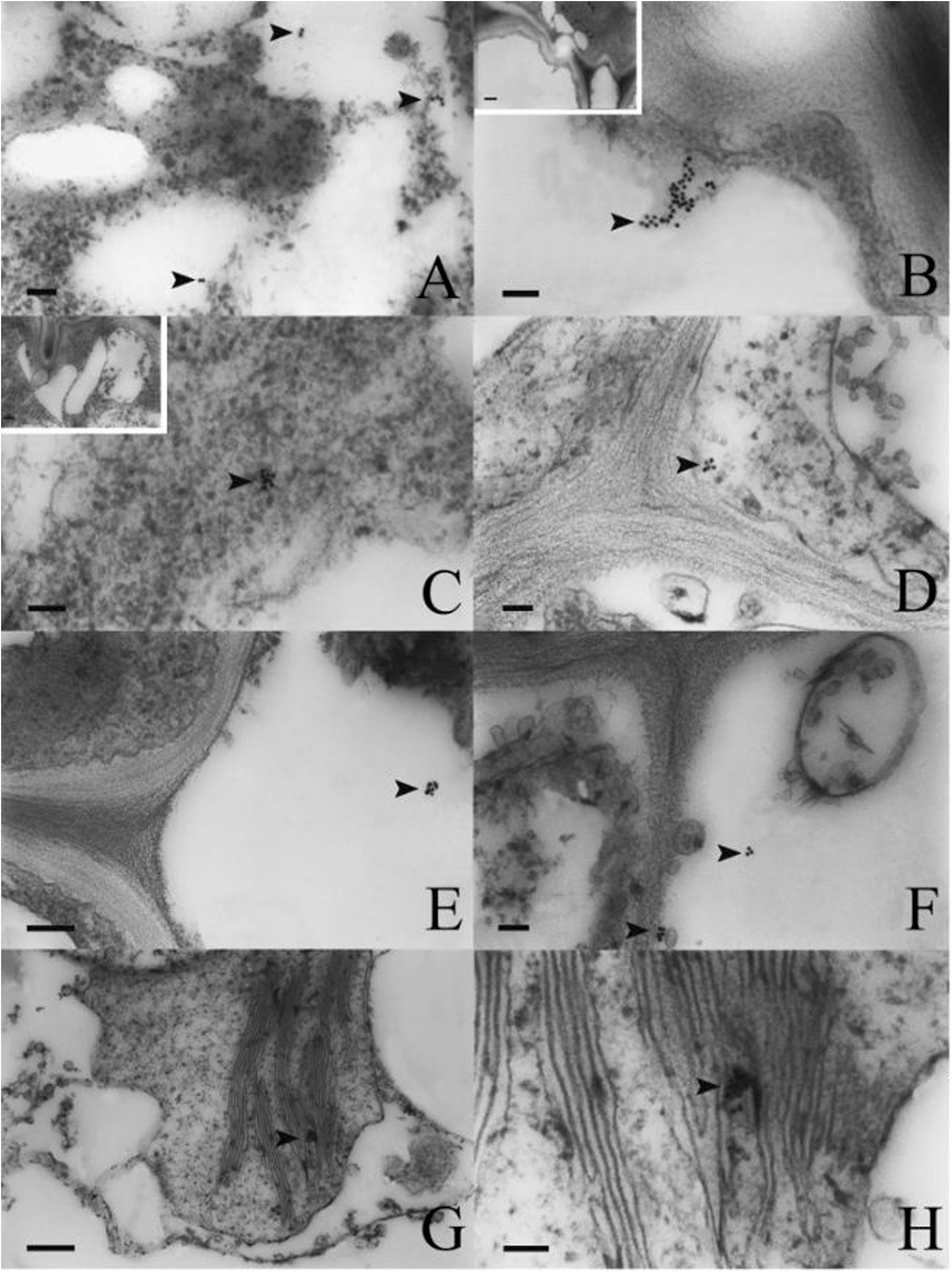

Supplement: Supplementary file 4 — Authors’ original file for figure 4 [file 40529_2013_25_MOESM4_ESM.tif]

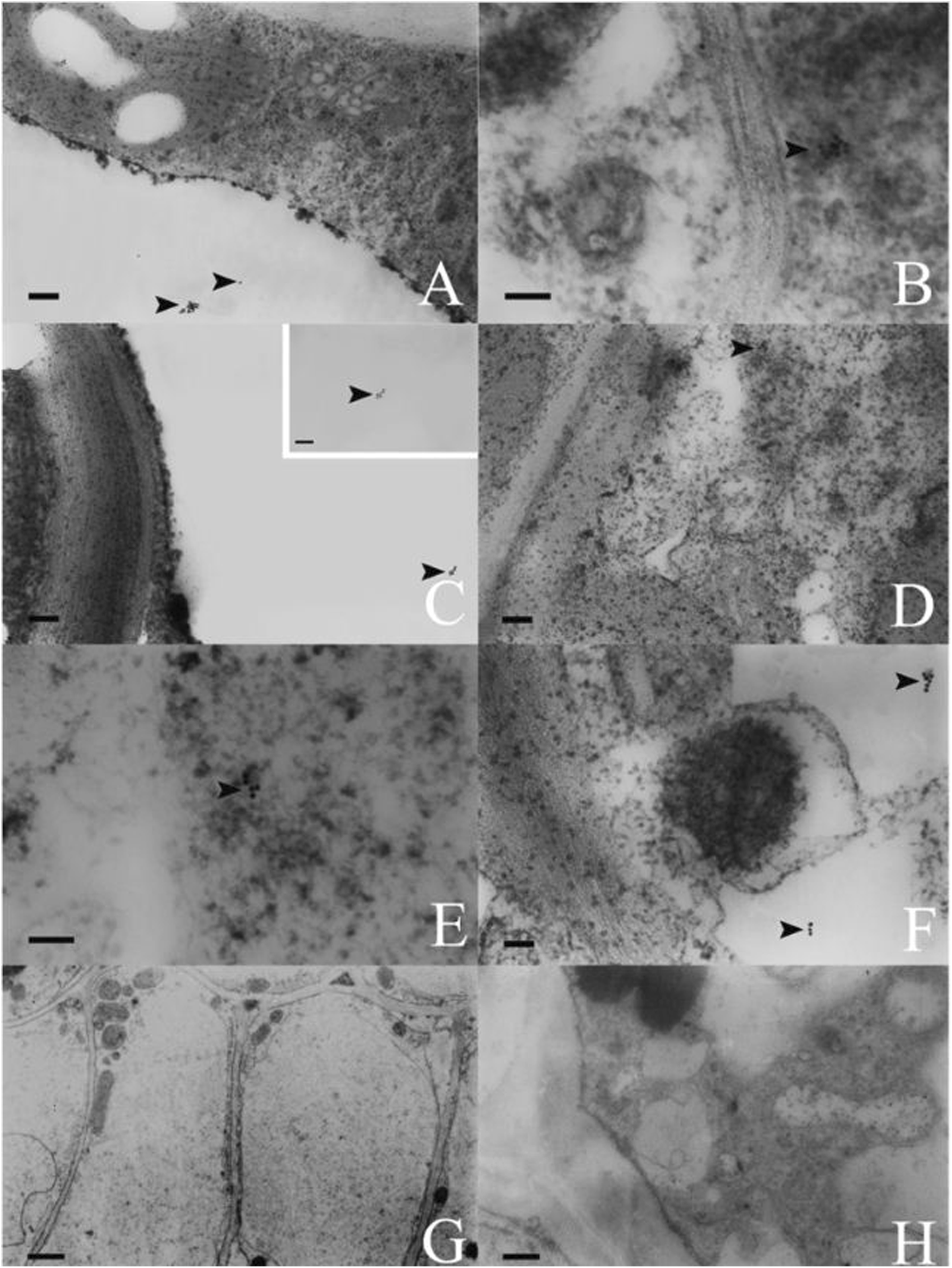

Supplement: Supplementary file 5 — Authors’ original file for figure 5 [file 40529_2013_25_MOESM5_ESM.tif]
